# Three-Dimensional Au/Ag Nanoparticle/Crossed Carbon Nanotube SERS Substrate for the Detection of Mixed Toxic Molecules

**DOI:** 10.3390/nano11082026

**Published:** 2021-08-09

**Authors:** Haonan Wei, Zhisheng Peng, Cheng Yang, Yuan Tian, Lianfeng Sun, Gongtang Wang, Mei Liu

**Affiliations:** 1 School of Physics and Electronics, Shandong Normal University, Jinan 250358, China; 2018020530@stu.sdnu.edu.cn (H.W.); chengyang@sdnu.edu.cn (C.Y.); 2018020524@stu.sdnu.edu.cn (Y.T.); 2 CAS Key Laboratory of Nanosystem and Hierarchical Fabrication, CAS Center for Excellence in Nanoscience, National Center for Nanoscience and Technology, Beijing 100190, China; pengzs2018@nanoctr.cn

**Keywords:** SERS, Au/AgNPs, 3D crossed CNTs, detection of complex solutions

## Abstract

Research on engineering “hotspots” in the field of surface-enhanced Raman scattering (SERS) is at the forefront of contributing to the best sensing indicators. Currently, there is still an urgent need to design a high-strength and large-scale electric field distribution method in order to obtain an ideal SERS sensor. Here, we designed a three-dimensional (3D) Au/Ag nanoparticle (NP)/crossed carbon nanotube film SERS substrate. The proposed structure formed by the simple preparation process can perfectly coordinate the interaction between the SERS substrates, lasers, and molecules. The denser “hotspots” can be induced and then distributed in holes enclosed by Au/AgNPs and the gaps between them. This process was verified by numerical simulations. The experimental results show that the proposed SERS substrate possesses an excellent sensitivity of 10^−12^ M (rhodamine 6G (R6G)), an enhancement factor of 1.60 × 10^9^, and a good signal reproducibility (the relative standard deviation is ~6.03%). We further use a Au/AgNP/crossed CNT substrate to detect complex solutions composed of toxic molecules, which shows that our proposed SERS substrate has a wide range of application potentials, especially in food safety.

## 1. Introduction

Surface-enhanced Raman scattering (SERS) with unique fingerprint recognition characteristics and nano-scale surface structures has been used as a promising ultra-sensitive detection technique to identify single molecules in complex environments [1,2,3,4,5,6]. Thus, SERS has diverse applications in toxin sensing [7], environmental monitoring [8], food safety [9], clinical diagnosis [10] and cultural heritage [11,12]. Electromagnetic enhancement mechanisms (EMs) and chemical enhancement mechanism (CMs) were proposed to analyze sensitivity [13,14,15]. The intense local electric field enhancement (EM) acting on molecules driven by the localized surface plasmon resonance (LSPR) of metal nanostructures (“hotspots”) is widely regarded as the main source of amplification for this inelastic scattering cross section. The enhancement factor (*EF*) can reach 10^6^–10^8^ times for EM. The charge transfer between biomolecules and substrates (chemical enhancement) can also increase the *EF* by 10^2^ times. Two-dimensional (2D) nanomaterials or 1D carbon nanotubes (CNTs) can be used for the charge transfer between biomolecules and materials. The combination of metal nanostructures and 2D nanomaterials or 1D CNTs for an ideal SERS platform has attracted much attention. Such coupling nanostructures can not only combine EM and CM enhancements but also control the distance and density of the “hotspots”. Zhang et al. reported that a flat graphene surface was covered on metal nanoislands to bring the probe molecules close to the “hotspots” [16]. Sun et al. fabricated flat MoS_2_ film/AgNPs used as a stable SERS substrate [7]. Multi-layer stacked nanostructures were also fabricated and analyzed [17,18,19,20]. Two-dimensional (2D) nanomaterials can be used as nanogap (or nanospacer) to obtain lateral and vertical “hotspots”. However, most of the probe molecules can only be adsorbed on the top layer due to flat structure, which restrict more molecules from reaching the “hotspots”.

Nanostructures with many hollow spaces (such as nanobowls and nano holes) can be used to increase the absorption ability of the molecules and the electromagnetic coupling of the optics. Recently, inspired by transform optics, Zhang et al. prepared substrates with a AgNP/Au nanobowl array structure, which significantly increased the intensity and volume of electromagnetic field by using the curvature of space [21]. Xiao et al. prepared hollow nanocones, which extended the “hotspots” to the whole hollow space of the nanocones, greatly improved the Raman signal, and achieved the detection of various traits [22]. Lei et al. synthesized the quasi-optical cavity of vertical ZnO nanosheets as a SERS substrate [23]. However, most work focuses on the detection of one kind of probe molecule in a solution. In fact, some toxic molecules often exist in extremely complex substances mixtures. The substances with high concentration around the trace probe molecule, which reach the vicinity of the “hotspots” first, prevent the capture of the probe molecule by the “hotspots” and then limit the signal enhancement. Thus, how to design high-intensity and large-scale “hotspots” to allow molecules to be detected easier is an urgent problem in the field of SERS.

In this work, we fabricate a 3D bimetal Au/AgNP/crossed CNT substrate to detect a mixed solution with the multiple toxic molecules. One-dimensional CNTs possess larger specific surface areas, and the crossed CNTs provide many frameworks for the adsorption of Au/AgNPs. Additionally, many porous structures can be formed by the crossed and stacked CNT film to allow the probe molecules to be easily accessed. The denser “hotspots” can be induced and are distributed in the void enclosed by Au/AgNPs and the nanogaps between them, which have been demonstrated by finite element simulation using COMSOL Multiphysics software. A detection limit of ~10^−12^ M, an enhancement factor of 1.60 × 10^9^, and a good signal reproducibility with the relative standard deviation (RSD) of ~6.03% for the rhodamine 6G (R6G) model molecule were obtained using our proposed nanostructures. Finally, two different mixture solutions (milk with the melamine (10^−6^ M) solution and a mixture composed of the Sudan red I (10^−6^ M), Congo red (10^−6^ M), and MG (10^−9^ M)) were successfully detected. The proposed 3D Au/AgNP/crossed CNT film structure can perfectly coordinate the interaction among SERS substrate, laser, and molecules, which can be used to detect the mixed solution. Thus, our proposed SERS substrate has enormous application potentials in the food safety field.

## 2. Materials and Methods

### 2.1. The Fabrication Process of the Au/AgNPs/Crossed CNTs Film

**Figure 1:**
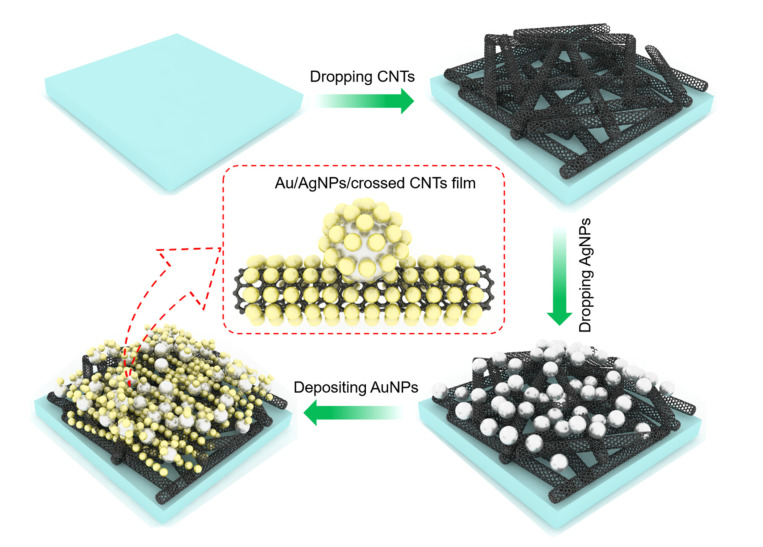
Schematic diagram of preparation of the Au/AgNP/crossed CNT substrate.

Figure 1 schematically illustrates the fabrication process of the Au/AgNP/crossed CNT film. First, all Si/SiO_2_ substrates with thicknesses of 500 µm and 300 nm were sonicated with acetone, alcohol, and deionized water (DI water) for 20 min and then treated with O_2_ plasma for 15 min. Second, CNT powder (0.01 g) was dispersed in a 10% sodium dodecyl sulfate solution (1 mL), maintained for 12 h to make the surface hydrophilic, and then washed with DI water. Such a modification facilitates the uniform dispersion of CNTs on the Si/SiO_2_ substrate. Subsequently, the CNT solution was dropped on Si/SiO_2_ substrates to form the crossed CNTs film. Third, the AgNP colloidal solution was synthesized according to the following method. The mixed solution of ethylene glycol (20 mL) and polyvinyl pyrrolidone (PVP, MW = 55,000, 0.25 g) was added to the open flask, and when the open flask was heated to 130 °C by an oil bath, a silver nitrate powder (0.05 g) was added to the mixed solution. After reacting for 1 h, the open flask containing the mixed solution was taken out of the oil bath, and acetone (60 mL) was mixed in the above solution and centrifuged at 12,000 rpm 3 times. The precipitate was cleaned and finally dispersed with deionized water (10 mL). The AgNPs were dopped on the crossed CNT film. Finally, the Au nanoparticles (AuNPs) was deposited on the AgNP/crossed CNT film surface by thermal evaporation. In detail, Au bulk (≥99.999%) was used and put into the molybdenum boat in thermal evaporation instrument with crystal oscillator, the vacuum pressure was below 10^−5^ Pa, the current was slowly increased to 90 amps, and the thickness parameter of Au was set to 3 nm. At this time, Au bulk was melted and was vaporized onto the AgNP/crossed CNTs film surface. Due to tension, liquid gold shrinks into AuNPs in the de-wetting process. According to the above process, the Au/AgNP/crossed CNT film (1#) was obtained. We also fabricated the AgNP/crossed CNT film/SiO_2_ (2#), the AuNP/crossed CNT film/SiO_2_ (3#), the AuNP/SiO_2_ (4#), the AgNP/SiO_2_ (5#), and the CNT film/SiO_2_ (6#) as control samples for the absorption spectra characterization, respectively. The manufacturing procedures of the control samples are as follows: first, the CNT solution was dropped on the SiO_2_ substrate to form the crossed CNT film/SiO_2_ (6#), and then, the prepared AgNP solution was dropped onto the crossed CNT film/SiO_2_ surface and then dried naturally. The AgNP/crossed CNT film/SiO_2_ (2#) was obtained. Similarly, the AuNPs were deposited on the crossed CNT film/SiO_2_ surface by thermal evaporation to obtain the AuNP/crossed CNT film/SiO_2_ (3#), while the AuNPs were deposited on the SiO_2_ surface by thermal evaporation to obtain the AuNP/SiO_2_ (4#) and the prepared AgNP solution was dropped onto the SiO_2_ surface and then dried naturally. AgNP/SiO_2_ (5#) was obtained.

### 2.2. Characterization

The surface morphology of the Au/AgNP/crossed CNT film was characterized by scanning electron microscopy (SEM, Sigma 500, ZEISS, Jena, Germany) equipped with X-ray energy spectrometer, and transmission Electron Microscope (TEM, JEM-2100F, Japan Electronics Corporation, Tokyo, Japan). A UV-vis Spectrophotometer (TU-1900, Shanghai Metash Instruments co., LTD., Shanghai, China) was used to characterize the absorption wavelength. An X-ray photoelectron spectroscopy instrument (XPS, PHI5300, Perkin-Elmer America, Shanghai, China) was used to obtain the compositions and chemical states. For SERS detection, the probe solution was dropped onto the surface of the SERS substrate and then air-dried. A Raman spectrometer (LabRAM, HR Evolution, Horiba, Paris, France) with 532 nm wavelength laser was used to analyze the SERS performance. The integration time was 4 s, and the laser power was 0.048 mW. The diffraction grid of 1800 gr/mm was chosen. The spot size was 2 μm, and a 50× objective (N.A. = 0.50) was used.

### 2.3. Simulation Calculations

In order to analyze the distribution of “hotspots” around the Au/AgNP/crossed CNT film, a finite element method simulation was performed through the COMSOL Multiphysics software (COMSOL Multiphysics 5.5, COMSOL, Stockholm, Sweden). Here, a laser with a wavelength of 532 nm was used as the excitation light to match the experiment condition. The light source type was considered a Gaussian beam, and the type of the Gaussian beam was chosen as the plane wave expansion to improve the convergence of the model. The direction of polarization and incident light was *x* and the negative *z* direction in all theoretical calculations, respectively. The perfect matching layer (PML) was set as the boundary condition in our simulations. The type of physics field interface was frequency domain electromagnetic wave. The size of the minimum mesh was 0.2 nm, and the type was free tetrahedral mesh. The refractive index of CNT, Ag, and Au were obtained from References [24,25,26], respectively.

## 3. Results and Discussion

### 3.1. Basic Characterization

**Figure 2:**
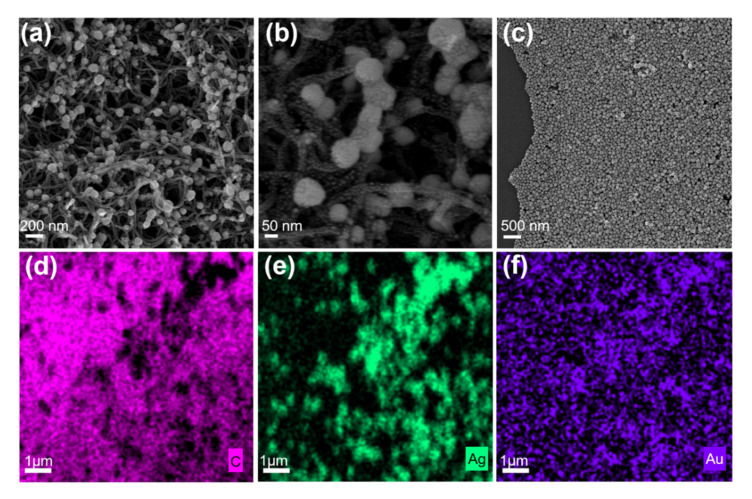
(**a**) Low-magnification and (**b**) high-magnification SEM images of the Au/AgNP/crossed CNT film. (**c**) SEM image of single-layer close-packed Au/AgNPs in the case without crossed CNT film. EDS elemental maps from (**d**) C, (**e**) Ag, and (**f**) Au on the Au/AgNP/crossed CNT film sample.

Figure 2a shows the crossed and porous morphology the morphology of the CNTs. The diameter of the CNT is 27.28 ± 3.53 nm (Figure S1d). The AgNPs were self-assembled on the crossed CNT film, and they were attached on the pipe wall of CNTs and are arranged from bottom to top around every hole. Figure S1a also shows the AgNPs that are also attached to CNTs. The diameter of AgNPs is ~84.24 ± 6.62 nm (Figure S1e). Figure 2b shows that the denser AuNPs with the average diameter of ~8.92 ± 1.13 nm (Figure S1f) were decorated on the surface of CNTs (Figure S1b) and AgNPs (Figure S1c). However, when there is no crossed CNT film, the single-layer close-packed Au/AgNPs were as presented in Figure 2c. Compared with the flat structure, the porous morphology effectively captures light and increases light utilization, which promotes the interaction between light and matter [27]. In order to further prove the composite structure of CNTs, AgNPs, and AuNPs, the local composition of the sample was also measured using an X-ray energy spectrometer. Figure 2d–f clearly reveal the existence of C (pink), Ag (green), and Au (purple) in the Au/AgNP/crossed CNT film. X-ray photoelectron spectroscopy analysis was carried out to analyze the elemental composition of the Au/AgNP/crossed CNT film quantitatively and qualitatively as shown in Figure S2. The C, Ag, and Au elements are all presented among the spectra, and the percentages of elemental Ag and Au contents are 31.95% and 9.14%. All of the characteristics strongly demonstrate that the Au/AgNP/crossed CNT film was successfully synthesized.

### 3.2. SERS Performance on Different Substrates

**Figure 3:**
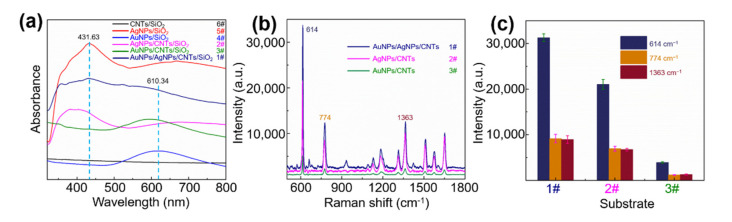
(**a**) Absorption spectra of these samples including Au/AgNP/crossed CNT film/SiO_2_ (1#), AuNP/crossed CNT film/SiO_2_ (3#), AgNP/crossed CNT film/SiO_2_ (2#), AuNP/SiO_2_ (4#), AgNP/SiO_2_ (5#), and CNT film/SiO_2_ (6#). (**b**) Raman spectra of R6G molecule (10^−5^ M) collected on the Au/AgNP/crossed CNT film (1#), the AgNP/crossed CNT film (2#), and the AuNP/crossed CNT film (3#). (**c**) The corresponding intensity distributions of the peaks at 614 cm^−1^, 774 cm^−1^, and 1363 cm^−1^ of the above three substrates.

In order to further verify the optical performance of the proposed SERS substrates, the absorption spectra of the Au/AgNP/crossed CNT film/SiO_2_ (1#), AuNP/crossed CNT film/SiO_2_ (3#), AgNP/crossed CNT film/SiO_2_ (2#), AuNP/SiO_2_ (4#), AgNP/SiO_2_ (5#), and CNT film/SiO_2_ (6#) were characterized in Figure 3a. For the AgNP/crossed CNT film and pure AgNP samples, there is a resonance peak at ~431.63 nm, which can be attributed to AgNPs. The resonance peak appearing at ~610.34 nm can be assigned to AuNPs in the AuNP/crossed CNT film and the AuNP samples. It is worth noting that the above two peaks can be observed in the absorption spectrum of the Au/AgNP/crossed CNT film sample. Due to the stronger light-trapping ability of the porous structure, a wider absorption is presented, which makes it easy to match the light absorption wavelength with the excitation wavelength in Raman detection and to improve the SERS signal intensity and sensitivity. In order to further prove the SERS activity of Au/AgNP/crossed CNT film sample, Raman detection of R6G molecule (10^−5^ M) was carried out on the Au/AgNP/crossed CNT film, the AgNP/crossed CNT film, and the AuNP/crossed CNT film, as shown in Figure 3b. Five random points on these three SERS substrates were chosen to be detected. The Raman spectra in Figure 3b were obtained from these three SERS substrates, respectively, while the height of the histogram in Figure 3c is the average value of five spectra for every characteristic peak (614 cm^−1^, 774 cm^−1^, and 1363 cm^−1^), and the error bars are fluctuations around the average. All fingerprint peaks of the R6G molecule are obviously observed, which exhibits an excellent signal-to-noise ratio that can be attributed to the crossed and porous morphology due to the existence of CNTs. In addition, there is also the contribution from CM of CNTs (Figure S3). The SERS spectrum intensity of every characteristic peak detected on Au/AgNP/crossed CNT film is higher than that on AgNP/crossed CNT film, but both of the above are much stronger than that on AuNP/crossed CNTs film. The coupling of AuNPs and AgNPs greatly promotes electric field enhancement and increases SERS intensity, which is discussed in the next section. Furthermore, for the AuNP/crossed CNT film substrate, the intensity is the lowest. Due to the stronger near-field coupling between particles and the sharper plasmon resonance peak, the SERS activity of AgNPs is better than that of AuNPs. Similarly, the Raman bands (D and G) of CNTs as the probe were all observed as shown in Figure S4. The phenomena that the plasmonic nanometals enhance the intensity of these bands are consistent with Figure 3b.

### 3.3. Theoretical Simulation Results of the Local Electric Field Distribution

**Figure 4:**
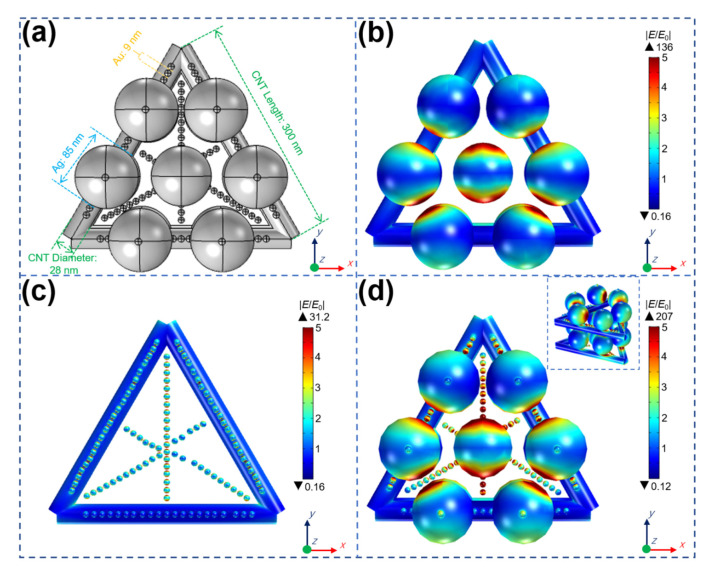
(**a**) Simulation setup of these substrates. COMSOL simulation of electromagnetic field distribution of (**b**) AgNP/crossed CNTs, (**c**) Au/crossed CNTs and (**d**) Au/AgNP/crossed CNTs.

In order to further analyze the best SERS performance of Au/AgNP/crossed CNT thin film substrate, commercial COMSOL software was used to calculate and analyze the local electric field distribution of these plasmonic structures. Figure 4a shows the size of each part of the structure. The diameter and length of the CNTs were about 28 nm and 300 nm, respectively. To provide a structure that is as similar as possible to the experimental results (Figure 2a), the crossed and stacked structure of CNTs were provided. AgNPs and AuNPs were distributed on the edge of the CNTs and inside the hole. Here, we only selected part of the Au/AgNPs to be arranged in an ordered structure. In the experiment, the metal nanoparticles were more densely distributed. Additionally, the diameters of Ag nanoparticle and Au nanoparticle were set to 85 nm and 9 nm, respectively. Compared with the local electric field distributed on AgNP/crossed CNTs and the local electric field distributed on AuNP/crossed CNTs (Figure 4b,c), in Figure 4d, it is observed that the local electric field of the Au/AgNPs/crossed CNTs structure is stronger and that the distribution is wider. The high-intensity electric field is distributed not only between AuNPs and the AgNPs but also in the holes formed by crossed CNTs, which has a field strength enhancement factor of up to >207, more than ~1.52 times and ~6.63 times larger than that of on AgNP/crossed CNTs and AuNP/crossed CNTs, respectively. These results indicate that the porous structure can contribute to the optimal strength and distribution of “hotspots” and SERS enhancement signals.

### 3.4. SERS Performances of the Proposed Au/AgNP/Crossed CNT Film SERS Substrate

**Figure 5:**
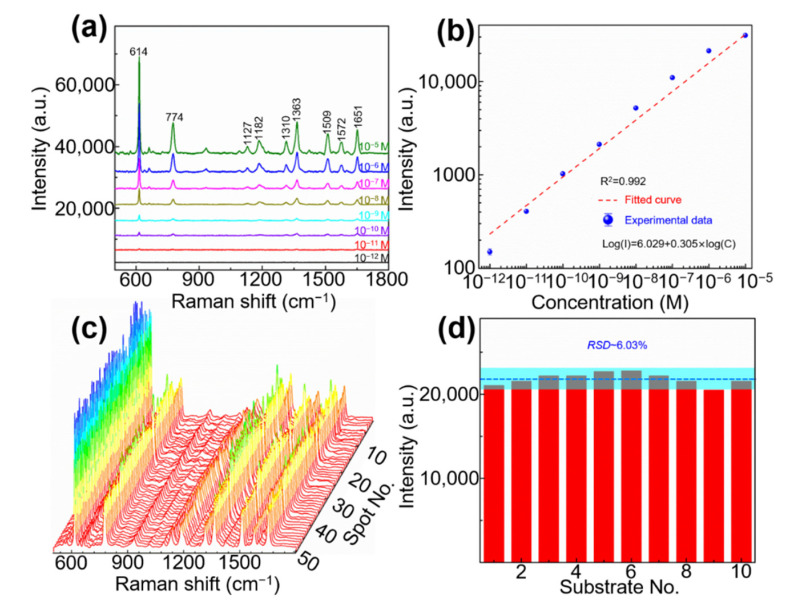
(**a**) SERS spectra of R6G molecules (concentration: 10^−12^–10^−5^ M) on the Au/AgNP/crossed CNT film substrate. (**b**) Linear relationship: relative intensities at 614 cm^−1^ as a function of the concentrations. (**c**) Spot-to-spot uniformity: SERS spectra of R6G (10^−6^ M) collected at 50 arbitrary positions on Au/AgNP/crossed CNT film. (**d**) Substrate-to-substrate reproducibility: relative intensities of the characteristic peak (614 cm^−1^) of SERS spectra of R6G (10^−6^ M) detected on 10 batch substrates.

After proving the optimal SERS performance of Au/AgNP/crossed CNT film, the sensitivity required as a SERS sensor is first investigated. As shown in Figure 5a, we detected the SERS spectra of R6G molecules (10^−12^–10^−5^ M) to obtain the limit of detection (*LOD*). All characteristic peaks at 614, 774, 1127, 1182, 1310, 1363, 1509, 1572, and 1651 cm^−1^ of R6G molecules with a concentration of 10^−5^ M to 10^−11^ M are detected. The vibrational mode of various peaks for R6G as shown in Table S1. The peak at 614 cm^−1^ is still easily identified when the concentration is 10^−12^ M, which indicates that the *LOD* of Au/AgNP/crossed CNT film as a SERS substrate is ~10^−12^ M. In order to obtain the enhancement factor (*EF*), the relative intensity of the characteristic peak at 614 cm^−1^ of R6G (10^−12^ M) on the SERS substrate is used as a reference, and the relative intensities of the characteristic peak at 614 cm^−1^ of R6G (10^−3^ M) on SiO_2_ is also used as a reference. Thus, the *EF* of the proposed Au/AgNP/crossed CNT film SERS substrate can be expressed as [28]:

(1) $$EF=\frac{I_{SERS}/C_{SERS}}{I_{RS}/C_{RS}},\;$$

where the SERS intensity (*I_SERS_*) is ~157.12 (Figure 5a). The normal Raman intensity (*I_RS_*) is ~98.12 according to previous report [29]. *C_SERS_* (10^−12^ M) and *C_RS_* (10^−3^ M) are the *LOD*s of the analytes detected on our SERS substrate and SiO_2_, respectively. Therefore, *EF* ≈ (157.12/10^−12^ M)/ (98.12/10^−3^ M) ≈ 1.60 × 10^9^. These results can be attributed to the crossed and porous morphology due to the existence of CNTs and the coupling of AgNPs and AuNPs that induces a large-scale electric field enhancement, which has been demonstrated by the theoretical simulation results. Therefore, once the probe molecules fall into “hotspots” in these pores, their SERS signal strength are greatly enhanced and easily identified. At the same time, CM also provides a good sensitivity and *EF*. The quantitative detection ability was characterized by changing the concentration of the R6G molecule and by recording the relative intensity of the characteristic peak at 614 cm^−1^. The log–log plot of the Raman intensity (*I*) of the characteristic peak at 614 cm^−1^ and R6G concentrations (*C*) follows an excellent linear relationship, as shown in Figure 5b. The linear equation is log(*I*) = 6.029 + 0.305 × log(*C*) (correlation coefficient: *R*^2^ = 0.992), where *I* and *C* are the SERS intensity and concentration of the probe molecule, respectively. The result indicates that the quantitative detection can be achieved based on the proposed Au/AgNP/crossed CNT film SERS substrate. In order to further verify the uniformity of each point signal and the repeatability of each batch of substrate signal, as shown in Figure 5c,d, Raman detection of R6G molecules (10^−6^ M) was performed, and 50 spectra were collected from 50 random points on a SERS substrate and 10 batches of Au/AgNP/crossed CNT film (one substrate for each batch). All data exhibit small fluctuations around average intensity and *RSD* [30,31]:

(2) $$RSD=\frac{\left|\Delta I\right|}{\bar{I}}\times 100\%=\frac{\left|I-\bar{I}\right|}{\bar{I}}\times 100\%,$$

where $\bar{I}$ is the average intensity and *I* is the collected relative intensity. Thus, ΔI is the maximum fluctuation surrounding by $\bar{I}$. As shown in Figure 5d, ΔI ≈ 1315.53 (a.u.), which is caused by the ninth batch substrate and $\bar{I}$ ≈ 21,804.53 (a.u.). Thus, the sample *RSD* can be obtained as 6.03%. These results demonstrate the acceptable uniformity and reproducibility of the proposed Au/AgNP/crossed CNT film SERS substrate. The many “hotspots” induced by the crossed and porous morphology (Figure 2a) may contribute to the outstanding uniformity. In addition, simple and easy-to-repeat preparation technology improves the excellent reproducibility. Therefore, our fabricated Au/AgNP/crossed CNT film SERS substrate has great potential for practical applications.

### 3.5. Practical Applicability Analysis of the Au/AgNPs/Crossed CNTs Film Substrate

**Figure 6:**
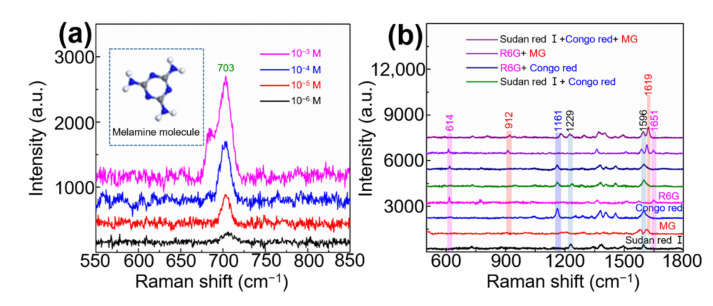
(**a**) SERS spectra of melamine solution in milk with a concentration of 10^−6^ to 10^−3^ M. (**b**) SERS spectra of Sudan red I (10^−6^ M, black line), malachite green (MG, 10^−9^ M, red line), Congo red (10^−6^ M, blue line), R6G (10^−10^ M, pink line), and their mixtures.

In order to study the practicability of Au/AgNP/crossed CNT film in human food safety, melamine was mixed with milk to obtain solutions with a concentration ranging from 10^−6^ to 10^−3^ M, and the SERS substrate was used as a sensor to detect the mixed solution. Melamine has been included in the list of carcinogens by the International Agency for Research on Cancer of the World Health Organization. The collected SERS spectra are shown in Figure 6a. The characteristic peak at 703 cm^−1^ belongs to the triazine deformation band, which can be clearly identified even for molecules with a concentration of 10^−6^ M. The detected concentration met the minimum requirement (0.15 mg/kg is approximately equal to 10^−5^ M.) of WHO. We further used the Au/AgNP/crossed CNT film SERS substrate to detect the more complex carcinogen solutions. The mixed solution of Sudan red I and Congo red; the mixed solution of R6G and Congo red; the mixed solution of R6G and MG; and the mixed solution Sudan red I, Congo red, and MG were detected as shown in Figure 6b. The fingerprint peaks of all molecules are sensitively detected. The peaks at 1229 cm^−1^ and 1596 cm^−1^, the peaks at 912 cm^−1^ and 1619 cm^−1^, the peaks at 1161 cm^−1^, and the peaks at 614 cm^−1^ and 1651 cm^−1^ are attributed to Sudan red I, MG, Congo red, and R6G molecule, respectively [32,33,34]. The sensitive detection is due to the crossed and porous morphology that easily allows the mixed molecules to enter, and the lots of volume “hotspots” greatly enhance the SERS signal of these molecules. Therefore, the proposed Au/AgNP/crossed CNT film shows great potential in detecting toxic molecules contained in human food.

## 4. Conclusions

In short, the Au/AgNP/crossed CNT film SERS substrate was successfully fabricated using a simple and low-cost method. Under laser excitation, the proposed plasmonic structure can produce high-intensity and wide-range electric field distribution, which was studied by theoretical simulation using COMSOL Multiphysics software. By choosing R6G as the probe molecule, we demonstrated the excellent sensitivity, quantitative detection capability, signal uniformity and reproducibility, and a high enhancement factor. Importantly, the excellent 3D SERS sensor was applied to detect the mixed solution of toxic molecules, which demonstrates that the SERS substrate we proposed has a wide range of application potentials, especially in the field of food safety.

## Supplementary Materials

The following are available online at https://www.mdpi.com/article/10.3390/nano11082026/s1, Figure S1: TEM images of the Au/AgNPcrossed CNT film and size distributions of CNTs, AgNPs, and AuNPs; Figure S2: XPS characterization of the Au/AgNP/crossed CNT film; Figure S3: Raman spectra of R6G molecules (10^−5^ M) detected on Si/SiO_2_, CNT/Si/SiO_2_, AuNP/Si/SiO_2_, AuNP/crossed CNT film, AgNP/Si/SiO_2_, and AgNP/crossed CNT film; Figure S4: Raman spectra of CNTs on the different SERS substrates; Table S1: the vibrational mode of various peaks for R6G.
