# Supplementary material for: Three-Dimensional Au/Ag Nanoparticle/Crossed Carbon Nanotube SERS Substrate for the Detection of Mixed Toxic Molecules
Source: Nanomaterials (Basel). 2021 Aug 9;11(8):2026. doi: 10.3390/nano11082026 (PMC8401542; doi:10.3390/nano11082026)
Supplement: Supplementary file 1 [file nanomaterials-11-02026-s001.zip › nanomaterials-1314883-supplementary.pdf]

nanomaterials

MDPI

Supplementary Materials

Three-Dimensional Au/Ag Nanoparticle/Crossed Carbon Nanotube SERS Substrate for the Detection of Mixed Toxic Molecules

Haonan Wei [1,2], Zhisheng Peng [2], Cheng Yang [1], Yuan Tian [1], Lianfeng Sun [2,*], Gongtang Wang [1,*], and Mei Liu [1,*]

[1] School of Physics and Electronics, Shandong Normal University, Jinan 250358, China; 2018020530@stu.sdnu.edu.cn (H.W.); chengyang@sdnu.edu.cn (C.Y.); 2018020524@stu.sdnu.edu.cn (Y.T.)

[2] CAS Key Laboratory of Nanosystem and Hierarchical Fabrication, CAS Center for Excellence in Nanoscience, National Center for Nanoscience and Technology, Beijing 100190, China; pengzs2018@nanoctr.cn

* Correspondence: slf@nanoctr.cn (L.S.); wanggt@sdnu.edu.cn (G.W.); liumei@sdnu.edu.cn (M.L.)

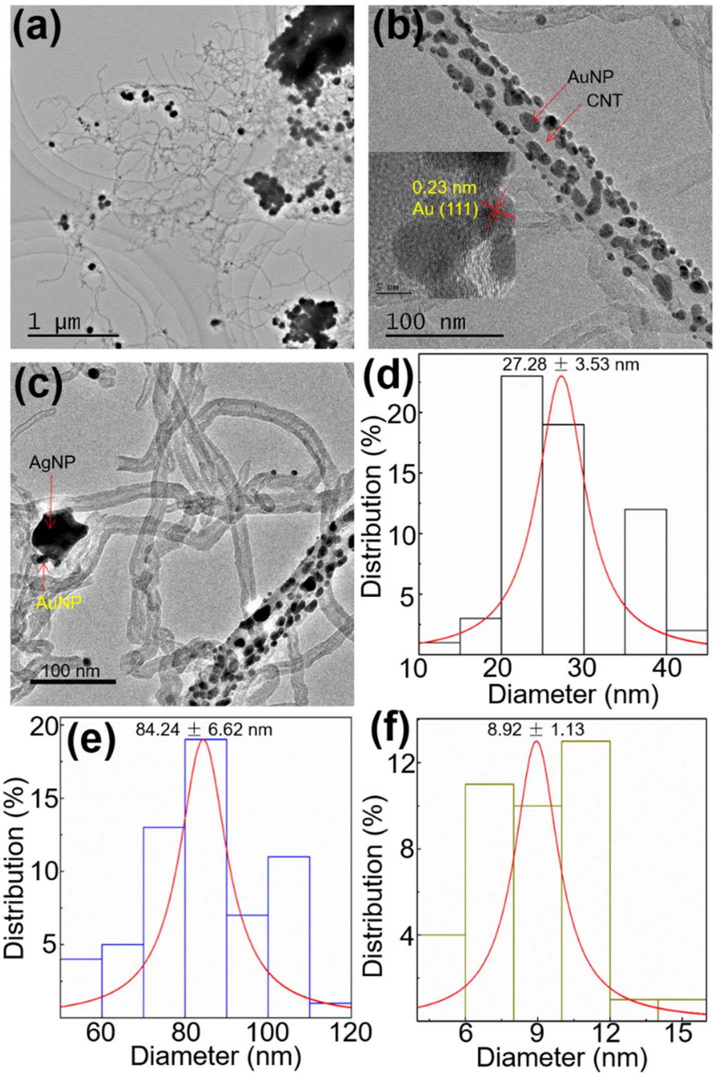

Figure S1. (**a**), (**b**) and (**c**) TEM images of the Au/AgNPs/crossed CNTs film. (**c**) Diameter distribution of (**d**) CNTs, (**e**) AgNPs and (**f**) AuNPs, respectively.

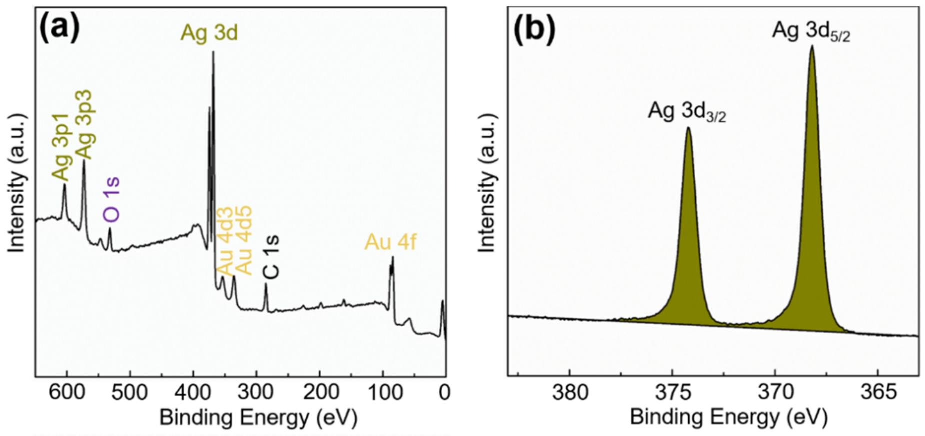

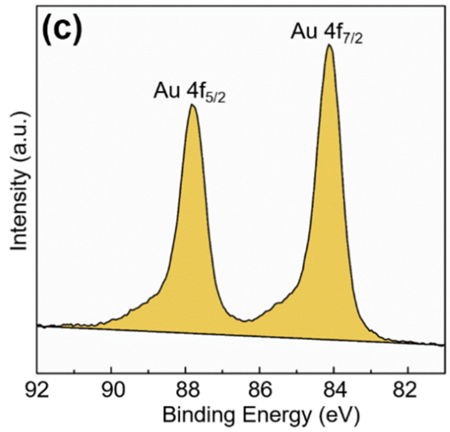

(d)

Element	FWHM (eV)	Atomic (%)
Ag3d	0.87	31.95
Au4f	0.8	9.14
C1s	1.61	58.91

Figure S2. XPS spectra of (**a**) survey, (**b**) Ag 3d, (**c**) Au 4f of Au/AgNPs/crossed CNTs film, (**d**) the corresponding percentage of element content.

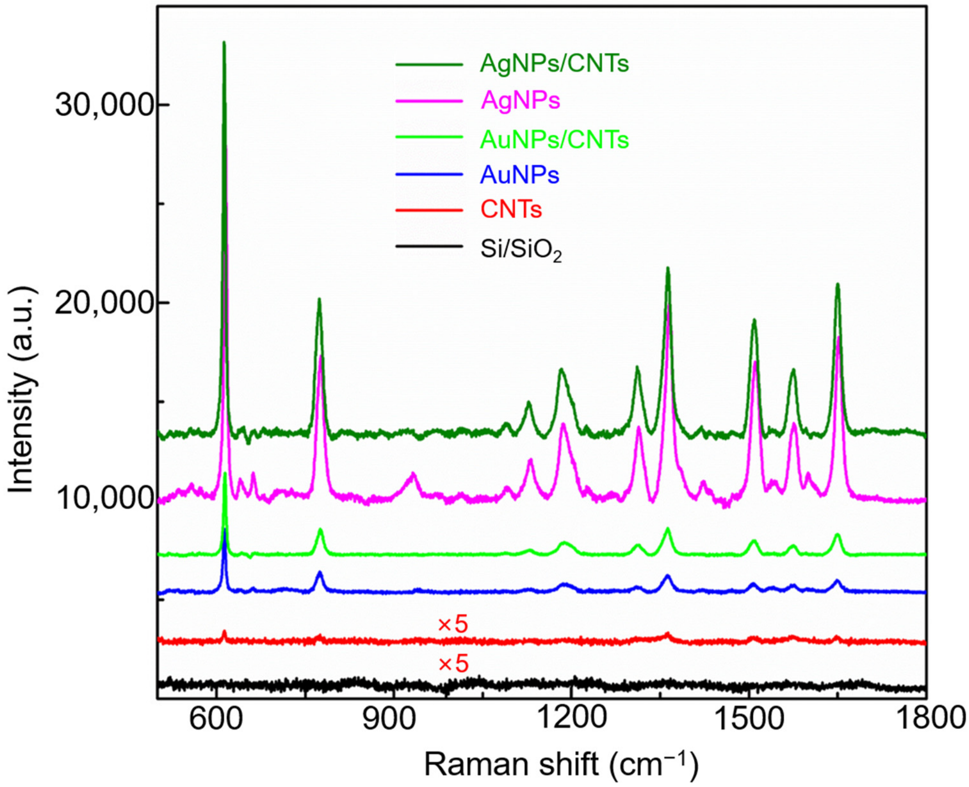

Figure S3. Raman spectra of R6G molecules (10^{-5} M) detected on Si/SiO_2, $CNTs/Si/SiO_2$, $AuNPs/Si/SiO_2$, AuNPs/crossed CNTs film, $AgNPs/Si/SiO_2$, AgNPs/crossed CNTs film.

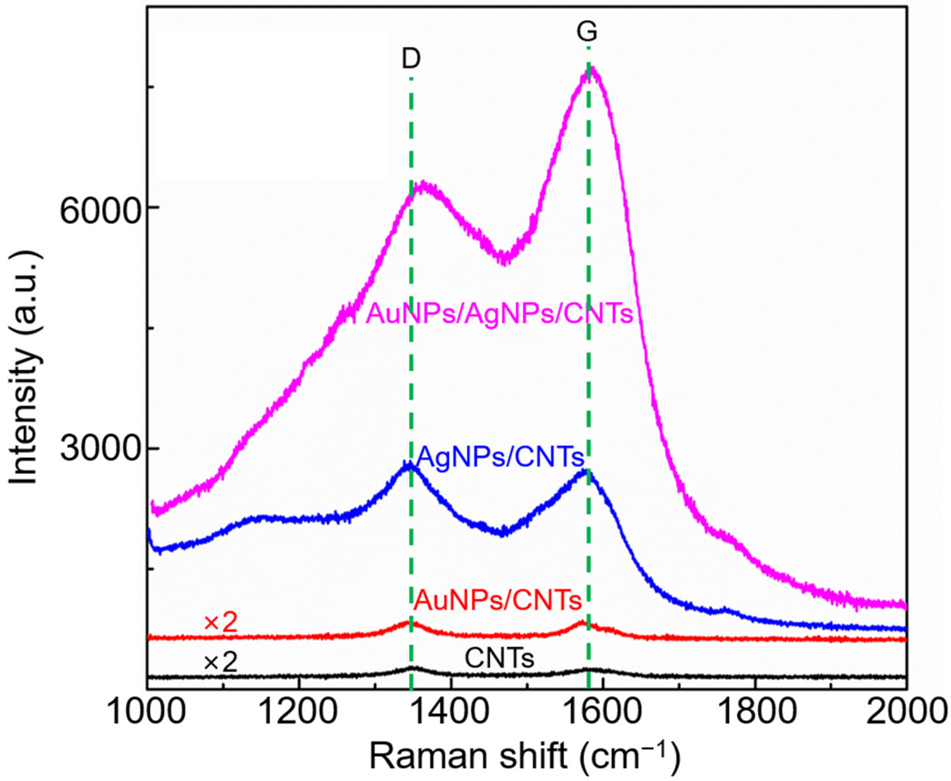

Figure S4. Raman spectra of CNTs on the crossed CNTs film, AuNPs/crossed CNTs film, AgNPs/crossed CNTs film and Au/AgNPs/crossed CNTs film.

Table S1. The vibrational mode of various peaks for R6G.

Raman shifts (cm^{-1})	Vibrational mode
614	C-C-C ring in-plane bending mode
774	C-H out-of-plane bend-ing mode
1182	C-H in-plane bending mode
1310	aromatic C-C stretching mode
1363	
1509	
1651	
